# Gestational Transient Thyrotoxicosis Complicated by Thyroid Storm in a Patient With Hyperemesis Gravidarum

**DOI:** 10.1210/jcemcr/luad064

**Published:** 2023-06-30

**Authors:** Camila A Villavicencio, Alberto Franco-Akel, Regina Belokovskaya

**Affiliations:** Department of Medicine, NYC Health and Hospitals Metropolitan / New York Medical College, New York, NY 10029, USA; Division of Endocrinology, NYC Health and Hospitals Metropolitan / New York Medical College, New York, NY 10029, USA; Division of Endocrinology, NYC Health and Hospitals Metropolitan / New York Medical College, New York, NY 10029, USA

**Keywords:** gestational transient thyrotoxicosis, hyperemesis gravidarum, thyrotoxicosis, thyroid storm

## Abstract

We describe a patient with gestational transient thyrotoxicosis (GTT) associated with hyperemesis gravidarum (HG) in a twin gestation complicated by thyroid storm resulting in intrauterine fetal demise. GTT is a well-documented complication of the first trimester of pregnancy that may affect up to 60% of pregnancies with HG. Typically, GTT is not associated with unfavorable maternal or fetal outcomes and has a spontaneous resolution. Aside from supportive care, the use of antithyroid drugs (ATD) in GTT is not regularly recommended. Although to our knowledge one similar case has been reported in the literature, the incidence of thyroid storm in GTT has not yet been established. Our case highlights that, although rare, GTT may be complicated by thyroid storm resulting in adverse maternal and fetal outcomes. Our case further suggests that consideration of ATD use in GTT should be given in pregnancies with higher serum human chorionic gonadotropin (hCG) concentration as occurs in HG and multiple pregnancies.

## Introduction

Gestational transient thyrotoxicosis (GTT) is a condition limited to the first half of pregnancy characterized by hyperthyroxinemia with an elevated free thyroxine (FT4) and suppressed serum thyroid stimulating hormone (TSH) [1]. It affects up to two-thirds of pregnancies complicated with hyperemesis gravidarum (HG) [1-3]. Both conditions are associated with higher human chorionic gonadotropin (hCG) concentrations as seen in multiple pregnancies [1, 2, 4-7]. GTT is not usually associated with unfavorable maternal and fetal outcomes [2]. Because of the short course and spontaneous resolution of GTT, the use of antithyroid drugs (ATD) is not routinely recommended [1-3, 6-9]. The incidence of thyroid storm in the setting of GTT is unknown [3]. We describe a case of GTT complicated by thyroid storm in a primigravid 20-year-old patient with twin gestation and HG.

## Case Presentation

A 20-year-old African American woman with history of asthma who was at 14 weeks with twin gestation (gravida 1, para 0) was brought by her family to the emergency department for altered sensorium and worsening lower limb weakness. At week 9 of the current pregnancy, she was diagnosed with HG requiring 6 days of inpatient management. That admission was complicated by an episode of supraventricular tachycardia which resolved with adenosine intravenously once. Laboratory testing included a TSH <0.01 uIU/mL (<0.01 mIU/L) [0.350–4.8 uIU/mL], FT4 3.6 ng/dL (46.3 pmol/L) [0.9–1.9 ng/dL], and hCG 124 301 mIU/mL [0.0–5.0 mIU/mL]. Given that her pre-pregnancy TSH was 0.99 uIU/mL, GTT was suspected. No specific management aside from supportive care was recommended. For 4 days prior to current presentation, the patient was noted to have increased somnolence, poor oral intake, worsening nausea, and emesis. No fever, chills, chest pain, shortness of breath, or diarrhea were reported. The patient's heart rate was 169 beats per minute, respiratory rate of 26 breaths per minute, and a normal body temperature of 97.5 °F. The patient was unable to provide coherent verbal information, and disorientation, mild agitation, hyperacusis, and mild generalized abdominal tenderness were noted. No exophthalmos, thyromegaly, or pretibial myxedema were found. Laboratory testing revealed serum glucose of 258 mg/dL (14.32 mmol/L) [70–99 mg/dL], white blood cell count 12 × 10^3^/mcL (12 × 10^9^/L) [4.3–11.0 × 10^3^/mcL], platelets 507 × 10^3^/mcL (507 × 10^9^/L) [150–450 × 10^3^/mcL], hemoglobin 14.2 g/dL (142 g/L) [12.0–16.0 g/dL], potassium 3.2 mEq/L (3.2 mmol/L) [3.5–5.1 mEq/L], bicarbonate 16 mmol/L (16 mEq/L) [22.0–29.0 mmol/L], creatinine 1.5 mg/dL (0.132 mmol/L) [0.7–0.9 mg/dL], lactic acid 5.1 mmol/L (45.9 mg/dL) [0.6–1.4 mmol/L], alanine aminotransferase 365 U/L (6.08 ukat/L) [0–31 U/L], aspartate aminotransferase 97 U/L (1.61 ukat/L) [0–32 U/L], lipase 175 U/L (2.9 ukat/L) [13–60 U/L], creatinine kinase 253 U/L (4.2 ukat/L) [20–180 U/L], hCG level 99 080 mIU/L, and negative SARS-CoV-2 PCR testing. Electrocardiography showed sinus tachycardia and a prolonged QTc of 473 milliseconds. A transvaginal ultrasound demonstrated absence of fetal heartbeat and fetal movement in either fetus. Given the suspicion for GTT on her last admission, new thyroid function studies were obtained and showed a TSH <0.01 mIU/L (<0.01 mIU/L), FT4 3.3 ng/dL (42.4 pmol/L), and triiodothyronine (T3) 239 ng/dL (367.1 nmol/L) [80–200 ng/dL]. TSH receptor antibodies (TRAb), thyroid peroxide antibodies, and antithyroglobulin antibodies were obtained. A Burch-Wartofsky point scale of 45 warranted admission to the medical intensive care unit for thyroid storm management. In addition to severe dehydration secondary to HG, sepsis was also considered as another precipitating event for thyroid storm. Blood cultures were obtained in the setting of suspected sepsis.

## Treatment

Intravenous fluids, methimazole, Lugol's solution, hydrocortisone, propranolol, cholestyramine, and broad-spectrum antibiotic therapy with ampicillin/sulbactam and vancomycin were initiated. After 48 hours, clinical and biochemical improvement were evident. Repeated thyroid function tests resulted in FT4 2.1 ng/dL (27 pmol/L) and T3 114 ng/dL (175.1 nmol/L). Lugol's solution and cholestyramine were stopped. TRAb, thyroid peroxide antibodies, and antithyroglobulin antibodies were negative. Thyroid ultrasound with doppler did not show hypervascularity nor thyroid nodules. These findings ruled out thyrotoxicosis of autoimmune origin, toxic multinodular goiter, and solitary toxic adenoma. On day 5, due to her biochemical euthyroid status with a FT4 1.10 mIU/L (14 pmol/L) and T3 83 ng/dL (127.5 nmol/L), ATD and beta-blocker were discontinued. Blood cultures were positive for methicillin sensitive *Staphylococcus aureus* suggesting a spontaneous septic abortion as the sepsis source. Dilation and evacuation were performed, and antibiotic therapy was switched to cefazolin.

## Outcome and Follow-up

The patient was discharged from the hospital on day 11 on oral cefadroxil for a 4-week completion of antibiotic therapy. At the 4-week follow-up, a new test result of TSH 0.544 uIU/mL (0.544 mIU/L) and FT4 1.02 ng/dL (14 pmol/L) were obtained. A year after hospital discharge a TSH 0.440 uIU/mL (0.440 mIU/L) and FT4 1.2 ng/dL (15.4 pmol/L) were found. The patient remains clinically and biochemically euthyroid without ATD. Patient has been counseled on the importance of birth control if not planning for pregnancy, to continue following up with Gynecology and to notify Endocrinology if she becomes pregnant.

## Discussion

GTT is classically a temporary complication of the first trimester of pregnancy that is characterized by hyperthyroxinemia with an elevated FT4 and suppressed serum TSH that results from the overstimulation of the TSH receptor by the alpha-subunit of hCG similar to that of TSH [1, 4, 6-8]. This TSH-like effect leads to an increased production of T4 and T3 levels and a subsequent lower TSH level [4-6, 8].

GTT can be seen in states with significantly elevated hCG concentrations as occurs in multiple pregnancies, gestational trophoblastic disease, and HG [2, 4-7]. It may also occur in pregnant women without exaggerated hCG production but whose TSH receptors carry genetic mutations that make them particularly hypersensitive to hCG [2, 6].

GTT affects up to 60% of pregnancies complicated by HG defined as severe nausea and vomiting in early pregnancy with more than 5% of weight loss, dehydration, and ketonuria [1-3]. Both conditions are usually transient and resolve spontaneously as hCG levels decline after reaching the peak at around 8 to 15 weeks of gestation [2, 7, 8]. Levels of hCG correlate with the degree of symptoms [3, 6, 7]. Patients with HG complicated by GTT may present with nausea, vomiting, weight loss, tachycardia, fine tremors, and mild proximal weakness at week 4 to 9 of gestation, although these clinical manifestations are not always evident and GTT may go unrecognized without an assessment of thyroid function [2, 8].

Rarely, thyrotoxicosis may present for the first time in pregnancy, with an incidence of 0.5 per 1000 pregnancies [9]. Diagnosing thyrotoxicosis during pregnancy is challenging due to associated physiological adaptations in maternal thyroid function [4]. These include an increased production of thyroid binding globulin mediated by estrogen, hCG stimulation of the TSH receptor in the thyroid gland, and the metabolism of T3 and T4 via increased activity of the type 3 iodothyronine deiodinase in the placenta [4]. Identifying the underlying etiology for thyrotoxicosis is key in the management [1, 6]. Graves’ disease (GD) and GTT are the most common causes of thyrotoxicosis [1, 8]. The prevalence of GD during pregnancy ranges from 0.2% to 0.5% [5, 8]. In contrast, GTT occurs in 2% to 11% of all pregnancies with a higher prevalence in Asian populations [2, 3, 5, 7, 8]. In both conditions, symptoms such as palpitations, anxiety, tremor, and heat intolerance are commonly found [1]. Moreover, differentiating GTT from Graves’ disease (GD) in a patient with no evidence of hyperthyroidism pre-gestation or stigmata of GD such as goiter or exophthalmos, may be difficult [2, 7, 8]. On average, serum hCG is higher in GTT than in GD; however, its clinical usefulness is limited [1]. When the diagnosis is unclear, measurement of TRAb can help distinguish between both entities [1, 2]. Nonetheless, physiological immunosuppression during pregnancy may suppress TRAb levels and its measurement be undetectable in patients with new-onset GD [2]. Comparative follow-up testing may be required [2].

Because GTT is transient and ATD are teratogenic during the first trimester of pregnancy, as opposed to GD, treatment with ATD is not regularly recommended in GTT aside from supportive care with hydration, anti-emetics, and electrolyte replacement [1-3, 6-9]. Literature suggests that ATD should be reserved for patients with severe GTT as the risk to the mother and fetus from overt hyperthyroidism outweighs the risk to the fetus from exposure to ATD [1, 7]. Remarkably, obstetrical outcomes have not improved in isolated cases in which GTT was treated with ATD [1].

In summary, thyroid storm is a rare complication of hyperthyroidism and entails a high mortality rate of 10% to 30% if not recognized immediately and aggressively treated [10]. Although the incidence of thyroid storm in the setting of GTT is unknown, studies suggest that patients with hyperthyroidism during pregnancy are tenfold more likely to develop thyroid storm [3, 10]. Our case illustrates how critical it is to determine the etiology of hyperthyroidism in early pregnancy, proper counseling on early identification of alarm symptoms, and it serves as an example of how GTT-associated thyroid storm may present. Early diagnosis and appropriate management are essential to prevent its associated potential fatal maternal and fetal events such as miscarriage, intrauterine fetal demise, preterm birth, low birth weight, intrauterine growth restriction, preeclampsia, and maternal congestive heart failure [1, 4, 5, 10].

## Learning Points

- GTT can be seen in states with significantly elevated serum hCG concentrations as occurs in multiple pregnancies and HG.
- Although uncommon, GTT may be associated with unfavorable maternal or fetal outcomes.
- The incidence of thyroid storm in GTT associated with HG is unknown but it is important to consider it as a potential complication of GTT.
- Aside from supportive care, a severe GTT presentation warrants the consideration of ATD.

## Data Availability

Original data generated and analyzed during this study are included in this published article.

## Abbreviations

ATD: antithyroid drugs
FT4: free thyroxine
GTT: gestational transient thyrotoxicosis
GD: Graves’ disease
HG: hyperemesis gravidarum
hCG: human chorionic gonadotropin
T3: triiodothyronine
T4: thyroxine
TRAb: thyroid stimulating hormone receptor antibodies
TSH: thyroid stimulating hormone
